# IMPROVED HEALTHSPAN AND LIFESPAN WITH LATE ONSET PHARMACOLOGICAL OR DIETARY INTERVENTIONS IN MICE

**DOI:** 10.1093/geroni/igz038.3207

**Published:** 2019-11-08

**Authors:** Sarah Mitchell, Michael MacArthur, Alice Kane, Margaret Torrence, Brendan Manning, James mItchell

**Affiliations:** 1 Harvard T.H. Chan School of Public Health, Boston, Massachusetts, United States; 2 Paul F. Glenn Center for Biology of Aging Research at Harvard Medical School, Boston, Massachusetts, United States

## Abstract

While late-onset dietary (e.g. calorie restriction) or pharmacological (e.g. rapamycin) interventions can extend longevity in rodents, whether or not they can be used to reverse or forestall onset of aging-related symptoms (i.e. frailty) remains untested. Here, we employed methionine restriction (MR), a dietary intervention associated with weight loss and longevity extension, or a fumagillin derivative, ZGN-1062, as a pharmacological agent associated with weight loss across multiple species. 21mo Male and female C57BL/6 mice were randomized to one of three groups: control, 0.1% MetR, or ZGN-1062 (1.5mg/kg). There was no difference in frailty index (FI) amongst groups at baseline (21 mo age). MetR and ZGN-1062 treatments resulted in modest weight loss independent of sex. After 6 months, FI increased consistent with reduced healthspan in control males (0.23+/-0.01 to 0.34+/-0.01 A.U, p<0.0001) and females at this age (0.19+/-0.03 to 0.24+/-0.01, p<0.0001). Male MetR and ZGN-1062 mice had significantly lower FI scores after 6 mo of treatment (MetR: 0.28+/-0.04, p<0.0001; ZGN-1062: 0.29+/-0.05, p=0.0002). While female mice were not significantly different from controls, they were, overall, less frail than males at 26 months, suggesting sexual dimorphism in the timing frailty onset in mice. Although the end points of this study have not been reached (survival studies are ongoing), the data obtained to date suggest that late-life treatments can improve healthspan markers, at least in male mice. Such treatments include a pharmacological intervention associated with weight loss, which may be a more practical therapeutic strategy towards mitigation of age-related healthspan decline than dietary restriction-based interventions.

